# Use of medications in women with triple-negative breast cancer between 2018 and 2019 in a Brazilian public hospital: a retrospective study

**DOI:** 10.1590/S2237-96222025v34e20240180.en

**Published:** 2025-02-21

**Authors:** Monique Cristine da Silva Pires, Mario Jorge Sobreira-da-Silva, Patrícia Portella de Araújo, Maely Peçanha Favero Retto

**Affiliations:** 1 Instituto Nacional de Câncer, Programa de Residência Multiprofissional em Oncologia, Rio de Janeiro, RJ, Brasil; 2 Instituto Nacional de Câncer, Coordenação de Ensino, Rio de janeiro, RJ, Brasil; 3 Instituto Nacional de Câncer, Serviço de Farmácia, Rio de Janeiro, RJ, Brasil

**Keywords:** Triple Negative Breast Neoplasms, Brazilian National Health System, Neoadjuvant Therapy, Chemotherapy, Adjuvant, Pharmacoepidemiology., Neoplasias de la Mama Triple Negativas, Sistema Único de Salud, Terapia Neoadyuvante, Quimioterapia Adyuvante, Farmacoepidemiología.

## Abstract

**Objective:**

To describe the profile of medication use in women with triple-negative breast cancer treated between 2018 and 2019 in a Brazilian public hospital.

**Methods:**

Descriptive and retrospective study, with data obtained from the Hospital Cancer Registry and physical and electronic medical records from a public hospital that is a reference in cancer treatment, in Rio de Janeiro. Descriptive analyses and analyses of time to treatment failure and overall survival were performed using the Kaplan Meier method.

**Results:**

Of the 176 patients, 39.0% were under 50 years of age and 47.7% were diagnosed at an advanced stage. Use of 12 chemotherapy regimens was identified, with neoadjuvant or adjuvant intent, for treatment of triple-negative breast cancer. The most commonly used treatment regimen included doxorubicin, cyclophosphamide and taxanes (docetaxel or paclitaxel). After 180 days, 76.1% of patients remained on the initial treatment. Average time until treatment failure was 7.6 months for those who followed the main regimen. Median overall survival was 34 months, and 55.7% of patients died by the end of the follow-up period (48 months).

**Conclusion:**

The results showed that treatment with doxorubicin, cyclophosphamide and taxanes (docetaxel or paclitaxel) was the most used in the patients analyzed, that average time to treatment failure using this regimen was less than one year and that more than half of the patients died within four years after diagnosis.

## Resumen

**Objetivo:**

Describir el perfil de uso de medicamentos en mujeres con cáncer de mama triple negativo tratadas entre 2018 y 2019 en un hospital público brasileño.

**Métodos:**

Estudio descriptivo y retrospectivo, con datos obtenidos del Registro Hospitalario de Cáncer y de registros médicos físicos y electrónicos de un hospital público de referencia en el tratamiento del cáncer, en Río de Janeiro. Se realizaron análisis descriptivos y análisis del tiempo transcurrido hasta el fracaso terapéutico y la supervivencia general mediante el método de Kaplan Meier.

**Resultados:**

De las 176 pacientes, el 39,0% eran menores de 50 años y el 47,7% fueron diagnosticadas en estadio avanzado. Se identificó el uso de 12 regímenes de quimioterapia, con finalidad neoadyuvante o adyuvante, para el tratamiento del cáncer de mama triple negativo. El régimen terapéutico más utilizado incluyó doxorrubicina, ciclofosfamida y taxano (docetaxel o paclitaxel). Después de 180 días, el 76,1% de las pacientes permanecía con el tratamiento inicial. El tiempo medio hasta el fracaso terapéutico fue de 7,6 meses para quienes siguieron el régimen principal. La mediana de supervivencia global fue de 34 meses y el 55,7% de las pacientes fallecieron al final del período de seguimiento (48 meses).

**Conclusión:**

Los resultados mostraron que el tratamiento con doxorrubicina, ciclofosfamida y taxano (docetaxel o paclitaxel) fue el más utilizado en las pacientes analizadas, que el tiempo promedio hasta el fracaso de este régimen terapéutico fue menor a un año y que más de la mitad de las pacientes murieron cuatro años después del diagnóstico.

**Ethical aspects**

This research respected ethical principles, having obtained the following approval data:

Research ethics committee: Instituto Nacional de Câncer

Opinion number: 5,970,438

Approval date: 28/3/2023

Certificate of submission for ethical appraisal: 67370523.6.0000.5274

Consent form: Exempt

## Introduction

Breast cancer is the most common type of cancer among women globally, accounting for approximately 23.8% in this population in 2022 ^1^. In Brazil, excluding non-melanoma skin cancer, it is the most common tumor, with a forecast of 74,000 new cases annually by 2025 ^2^.

Among breast cancers, triple-negative breast tumors, defined by the absence of hormone receptors and non-amplification of the human epidermal growth factor receptor-2 gene, are distinguished by their aggressiveness and unfavorable prognosis ^3^. This characterization makes it unsuitable for hormone therapy and targeted treatments, limiting treatment options, mainly to the use of systemic chemotherapy, which is a clinical challenge ^4^ ^,^ ^5^.

In the last decade, there has been significant progress in research aimed at developing new treatments for triple-negative breast tumors, with promising results using immunotherapy and targeted therapies ^6^ ^,^ ^7^ ^,^ ^8^. International guidelines highlight that, in order to achieve better outcomes, it may be necessary to combine innovative therapies with conventional systemic chemotherapy in different phases of initial treatment ^9^.

However, in Brazil, systemic chemotherapy continues to be the main option available for treating this neoplasm in the Brazilian National Health System (*Sistema Único de Saúde* - SUS) ^10^ ^,^ ^11^. Studies indicate that, in addition to the delay in adopting new technologies, restricted access to quality healthcare, late diagnosis and differences in the availability of innovative treatments contribute to variable outcomes between populations. Such disparities are even more evident in low- and middle-income countries, such as Brazil, where socioeconomic barriers worsen the challenges faced by patients ^12^ ^,^ ^13^ ^,^ ^14^.

Due to the heterogeneity of the characteristics of triple-negative breast tumors and the medications currently available on the SUS, establishing a standard treatment regimen is a challenge. Data on the procedures and protocols adopted to treat this neoplasm in Brazil are scarce. Therefore, this study aimed to characterize the profile of medication use in women with triple-negative breast tumors, with initial neoadjuvant and adjuvant intent, in order to understand the treatment standard adopted.

## Methods

### Design and background

This was a descriptive and retrospective study with patients diagnosed with triple-negative breast tumor, between January 2018 and December 2019, treated with chemotherapy with initial neoadjuvant and adjuvant intent, in a public hospital that is a reference for cancer treatment in Brazil.

### Participants, sample and eligibility criteria

The total population in the period consisted of 311 women over 18 years old. Taking a 5% sampling error and a 95% confidence interval (95%CI), a minimum sample of 138 patients in the study was estimated. The following patients were excluded: pregnant and breastfeeding women; those with concomitant or non-primary breast tumors; those who had received previous systemic treatment in another institution; those selected for research protocols; those whose physical medical records were not located; those who used hormone therapy or trastuzumab and those who had hormone receptor positivity or epidermal growth factor receptor-2 positivity at some point during treatment - despite initially being diagnosed with a triple-negative breast tumor.

### Data source and variables

The database used to identify the study population was the Hospital Cancer Record Integrator of the institution where the study was carried out. Subsequently, participants were selected based on information from physical and electronic medical records and the hospital administration system.

The variables collected were categorized into three axes: sociodemographic data (age less than 50 years old and age equal to or greater than 50 years old, family history of cancer, race/skin color, level of education and social habits); clinicopathological data (performance scale, histological subtype, histological grade, Ki-67 marker expression, clinical and pathological staging); therapeutic data (surgery, radiotherapy, treatment goal and chemotherapy regimens used - including doses, number of cycles performed and medications, in addition to reasons for changing therapeutic regimens and lines of palliative treatment, when applicable). Malignant tumor classification, known as TNM (tumor, lymph node, metastasis) ^15^, was used to define clinical and pathological staging. Those with clinicopathological stages 3B, 3C and 4 were considered to be at an advanced stage. In addition, date of death was collected in order to analyze overall survival.

### Data organization and analysis

The data were organized and analyzed using Microsoft 365 Excel® software. We calculated the frequency of categorical variables and measures of central tendency and dispersion of continuous variables. We analyzed the distribution of patients across multiple chemotherapy regimens used and reasons for discontinuing drug treatment. Total patient follow-up time was 48 months, based on the date of diagnosis.

The time (in months) for completion of the regimens used as first line was estimated using the initial and

final dates of treatment. Once this parameter was established, the transitions of the treatment lines were analyzed using Python software version 3.12.1, at 30, 60 and 180 days.

When identifying the most used regimen in the first line of initial therapy, we analyzed the average interval between the start and failure of treatment, over 24 months, using the Kaplan-Meier method

^16^. The following were considered failures: use of the subsequent line with palliative intent, death and exclusive palliative care. Overall survival at 48 months was estimated starting from the date of diagnosis. In cases where loss of follow-up occurred, censoring occurred at the time of the last clinical record held on the institution’s medical records. JAMOVI ® software version 2.3.28 was used for these analyses ^17^ ^,^ ^18^.

## Results

A total of 238 women were recruited, 62 of whom were excluded due to: lack of record of systemic treatment (n=24; 38.7%); showing hormonal positivity and/or using hormone therapy (n=9; 14.5%); history of systemic treatment at another institution (n= 8; 12.9%); history of previous chemotherapy for other tumors and/ or non-primary breast tumor (n=8; 12.9%); showing positivity for human epidermal growth factor 2 and/or use of trastuzumab (n=5; 8.1%); having concomitant tumors (n=4; 6.5%); participation in research protocols (n=3; 4.8%); and physical medical record not being found (n=1; 1.6%).

Of the 176 research participants, 85 (48.3%) were diagnosed in 2018. Median age was 54 (min=24; max=90) years, with 39.0% under the age of 50. Of the total, 77.8% reported being non-White and 50.6% only had elementary education. 

The most common tumor subtype was invasive ductal carcinoma (95.5%), and, at the time of diagnosis, the majority had performance status 1 (55.1%), histological grade type II (50.0%), Ki 67 ≥14% (97.0%) and advanced stage (47.7%). Of those who underwent surgery (n=136), 54.4% underwent radical mastectomy. A total of 67.6% underwent adjuvant radiotherapy. Table 1 shows the profile of the patients analyzed.

**Table 1:** Sociodemographic and clinical characteristics of patients with triple negative breast cancer undergoing treatment with neoadjuvant or adjuvant intent (n=176)

Variables	n (%)
**Family history of cancer**	
Yes	78 (44.3)
No	98 (55.7)
**Race/skin color**	
Non-White	137 (77.8)
White	39 (22.2)
**Schooling**	
Elementary education	89 (50.6)
High school education	65 (36.9)
Higher education	18 (10.2)
Information missing	4 (2.3)
**Age**	
18-49 years	67 (39.0)
≥50 years	109 (61.0)
Performance Status	
0	64 (36.4)
1	97 (55.1)
2	8 (4.5)
3	4 (2.3)
4	1 (0.6)
Information missing	2 (1.1)
**Histological subtype**	
Invasive ductal carcinoma	168 (95.5)
Invasive lobular carcinoma or others	8 (4.6)
**Histological grade**	
1	1 (0.6)
2	88 (50.0)
3	86 (48.8)
Information missing	1(0.6)
**Ki 67 marker expression.**	
>14%	170 (97.0)
<14%	6 (3.0)
**Staging**	
1A	7 (4.0)
2A	23 (13.1)
2B	31 (17.6)
3A	29 (16.5)
3B	75 (42.6)
3C	6 (3.4)
4	3 (1.7)
Information missing	2 (1.1)
**Surgery**	
Yes	136 (77.3)
No	40 (22.7)
**Type of surgery**	
Radical mastectomy	74 (42.0)
Segmentectomy	36 (20.5)
Simple mastectomy	25 (14.2)
Surgery performed in another hospital	1 (0.6)
Surgery not performed	40 (22.7)
**Radiotherapy**	
Yes	119 (67.6)
No	57 (32.4)

We identified 12 chemotherapy regimens, with neoadjuvant intent (10.8% of cases) or adjuvant intent (89.2% of cases), for the treatment of triple-negative breast tumors. The combination of doxorubicin, cyclophosphamide and taxane (docetaxel or paclitaxel) was the main treatment regimen used (80.7%). The least frequent regimens were doxorubicin (0.6%), docetaxel (0.6%) and the combination of fluorouracil, methotrexate and cyclophosphamide (0.6%).

**Figure 1.**
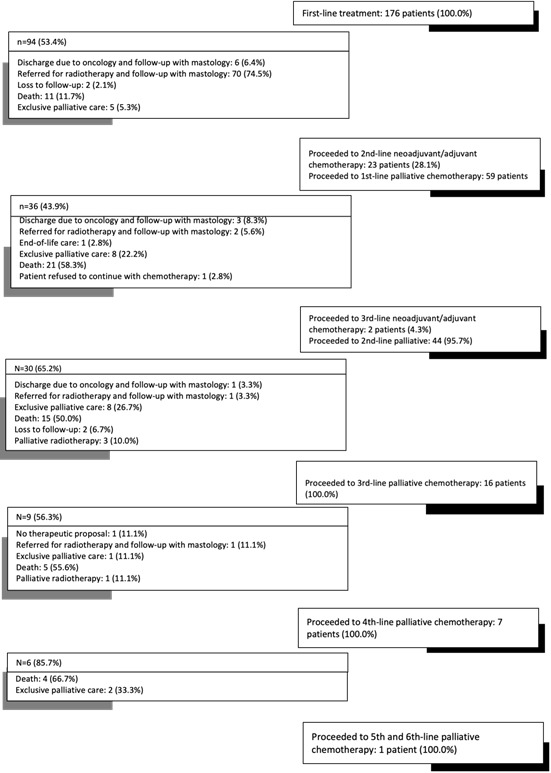
Distribution of patients with triple-negative breast cancer across multiple chemotherapy regimens and reasons for discontinuation of treatment with medication

In total, 23 patients proceeded to second-line chemotherapy with curative intent, being treated with: carboplatin and paclitaxel (43.0%), paclitaxel (26.0%), capecitabine (9.0%), carboplatin (4.4 %), cyclophosphamide and taxane (4.4%), gemcitabine and cisplatin (4.4%), docetaxel (4.4%) and fluorouracil, methotrexate and cyclophosphamide (4.4%). In the third neoadjuvant line, cisplatin was the drug chosen to treat two patients. The distribution of patients, alongsuccessive lines of chemotherapy, and the reasons for discontinuing treatment are shown in Figure 1 A.

The reasons recorded on the medical records for applying multiple regimens with neoadjuvant or adjuvant intent were: low therapeutic effectiveness; presence of large lesions that made it difficult to perform surgical procedures; clinical condition of the patient; and disease progression.

We found that, after using the first therapeutic regimen with curative intent, 82 (46.6%) participants continued using drug treatment. Of these, 71.9% showed disease progression and started using palliative chemotherapy. The reasons for discontinuation were: therapeutic response - patients were followed up by mastologists, in treatments that did not involve systemic chemotherapy; absence of therapeutic proposal or impeding clinical conditions - for these, it was decided to employ exclusive palliative care or end-of-life care.

**Figure 2.**
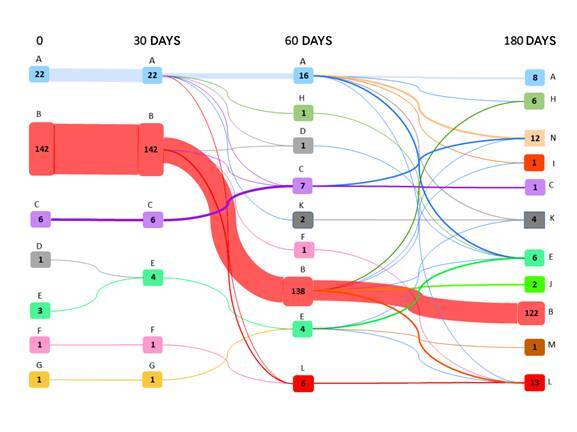
Sankey diagram with therapy changes, at 30, 60 and 180 days, in patients with triple-negative breast cancer

Legend: A: doxorubicin, cyclophosphamide; B: doxorubicin, cyclophosphamide and taxane; C: cyclophosphamide and taxane; D: cyclophosphamide, methotrexate and fluorouracil; E: paclitaxel; F: doxorubicin; G: docetaxel; H: carboplatin and paclitaxel; I: gemcitabine and cisplatin; J: capecitabine; K: death; L: palliative chemotherapy; M: loss to follow-up; N: non-drug therapy.Notes: The lines indicate departure from the origin and arrival at the destination; The number of patients is represented by numbers.

Figure 2 shows that, within 30 days after starting treatment, only one patient had switched to a second line of neoadjuvant treatment. We found that, within 60 days, 22 patients started using therapeutic regimens for palliative purposes, and two deaths were recorded. After 180 days, 76.1% of patients continued to undergo the initial treatment.

**Figure 3:**
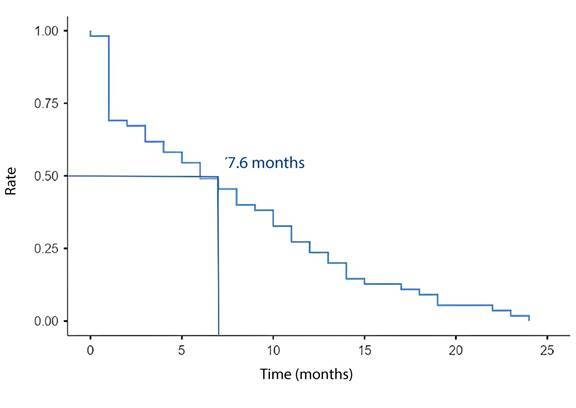
Analysis of temporality between the start of treatment and failure of the most used therapeutic regimen, consisting of doxorubicin, cyclophosphamide and taxane, in treatment of triple-negative breast cancer with neoadjuvant and adjuvant intent, over 24 months (n=55)

When analyzing treatment failure within 24 months, two patients were lost to follow-up. Of the remaining 174, 70 (40.2%) presented failures related to the following reasons: 55 (78.6%) used a subsequent therapeutic line for palliative purposes; 11 (15.7%) died; and four (5.7%) were referred to exclusive palliative care. Among the 70 patients, 55 (78.6%) had used the doxorubicin, cyclophosphamide and taxane regimen, with an average time to failure of 7.6 months, over 24 months (Figure 3). 

During the systemic treatment period, 56 patients died (31.8%). Additionally, 85 patients (48.3%) progressed to the non-systemic treatment phase. At the end of the follow-up period, we found that: 60 (70.6%) remained under mastology follow-up, without the need to return for additional systemic therapy; 15 (17.6%) died; and five (5.9%) needed to be redirected to systemic therapy. We also found that two (2.3%) participants had disease progression without indication of drug treatment, one (1.2%) was discharged and referred for follow-up at a primary care health service, one (1 .2%) died from COVID-19 and one (1.2%) was admitted to hospital with non-invasive support, with no record after hospitalization.

**Figure 4:**
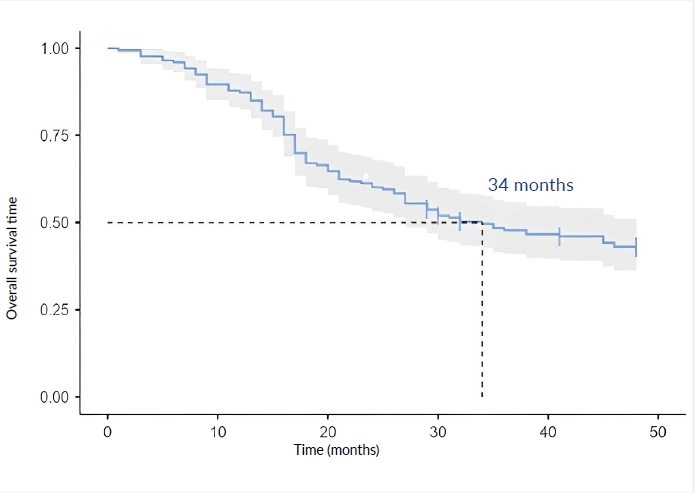
Analysis of overall survival analysis of women with triple-negative breast cancer over 48 months

We found that, after 48 months, all patients who had been referred for end-of-life care, exclusive palliative care, palliative radiotherapy, who chose not to continue with pharmacological treatment, or who for whom there was considered to be no theraputic proposal, died. Figure 4 shows overall survival at 48 months. Median survival of 34 months was found, and only 43.2% of the patients analyzed remained alive at the end of follow-up. Four patients were lost to follow-up at the end of the study, due to lack of clinical records covering a period greater than 24 months.

## Discussion

We found that the most used therapeutic regimen in the first line of treatment was the combination of doxorubicin, cyclophosphamide and taxane, and that up to three lines of treatment with curative intent were used. However, a significant proportion of patients required palliative treatment after the first line of curative treatment. Median time to treatment failure with doxorubicin, cyclophosphamide and taxane was 7.6 months, median overall survival was 34 months from diagnosis and, at the end of the follow-up period, less than half of the patients remained alive.

The analysis of the sociodemographic and clinical profile demonstrated similarity to the findings of a retrospective cohort conducted from June 2008 to January 2009, which analyzed women diagnosed in2001 (n=2,198) and 2006 (n=2,714) with different breast cancer subtypes in Brazil ^19^. However, it is worth highlighting the significant prevalence of young women, with low education levels and of non- White race/skin color, in the context in which cancer manifests itself as an issue of wide-reaching relevance, encompassing health, social and economic dimensions ^1^, since the triple-negative breast tumor is known to affect young women and has a poorer prognosis, compared to other types of breast tumors ^20^ ^,^ ^21^.

Recent studies indicate that poorer prognosis may not only be associated with the biological characteristics of the tumor. Different authors have investigated the influence of social disparities on the clinical outcomes of women with this type of tumor and demonstrated that non-White race/skin color, socioeconomic deprivation and social stress were associated with lack of access to healthcare and worse overall survival ^12^ ^,^ ^13^ ^,^ ^14^ ^,^ ^22^. This shows that socioeconomic aspects may be related to worse clinical outcomes in patients with triple-negative breast tumors.

The distribution of participants across progressive lines of treatment pointed to rapid progression of the disease after the first line of neoadjuvant and adjuvant treatment, and the need to begin palliative treatment. The data also reveal that, after an unsatisfactory response to the regimen composed of doxorubicin, cyclophosphamide and taxane, the combination of gemcitabine and cisplatin emerged as the most recurrent option, which is a preferred regimen for treatment of metastatic triple-negative breast tumor and is considered safe for patients treated with other lines of chemotherapy, when low doses are administered ^23^ ^,^ ^24^.

Analysis of the failure rate at 24 months for the therapeutic regimen comprised of doxorubicin, cyclophosphamide and taxane revealed considerable ineffectiveness after use of the first line of treatment. It is important to highlight that the therapeutic regimens used to treat the patients in this study were based on internal practices, aligned with the most up-to-date international recommendations for the treatment of triple-negative breast tumors. However, such regimes were dissonant with the diagnostic and therapeutic guidelines in force in 2018 in the Brazilian public health system ^10^, which recommended an even more restrictive therapeutic arsenal for the neoadjuvant or adjuvant treatment of triple-negative breast tumors. In this sense, it is possible to assume that the adoption of outdated therapeutic regimens, provided for in the guidelines, could result in even more unfavorable outcomes in the treatment of these patients.

Analysis of overall survival at 48 months demonstrated that, despite the increase in updates derived from international guidelines - which culminated in the most modern treatment in line with Ministry of Health recommendations -, what was offered to treat these women may not have been enough to obtain better outcomes, given other therapeutic possibilities. Or, furthermore, that treatment may be started by these patients at an inopportune time. Studies demonstrate that access to breast cancer treatment in Brazil’s public health system does not occur as recommended, and that difficulties can be exacerbated when it comes to the younger population ^25^ ^,^ ^26^.

A retrospective study that included 8,601 women and evaluated the role of chemotherapy in stage IA triple-negative breast cancer, between 2010 and 2019, demonstrated that use of chemotherapy was associated with improved patient survival. Considering that, in the present study, the majority of patients accessed specialized care with advanced-stage disease, more appropriate management of these patients by the health network, and the consequent early start of treatment, could culminate in more favorable outcomes.

As a suggestion, effective implementation of management strategies in the Brazilian public health system may result in the timely referral of patients to specialized care services, ensuring that the application of technologies already existing in the care network is sufficient to provide more effective treatment. Furthermore, the incorporation of new technologies should be considered, but not at the expense of optimizing referral flows and use of existing resources, in order to ensure that the use of more financially costly medications is done in an optimized and sustainable manner, since paying for them is challenging for health systems ^28^.

Regarding the study of medication use, certain limitations must be considered, including the use of data sources such as isolated medical prescriptions or medical records and the collection and consequent standardization of data on medications ^(29)^. With the aim of minimizing uncertainties about the data obtained from these data sources, our study compared prescription data and/or physical records with those recorded on the hospital’s computerized systems.

The results showed that the most used therapeutic regimen, in the neoadjuvant and adjuvant setting, was the combination of doxorubicin, cyclophosphamide and taxane, with an average time until treatment failure of 7.6 months, and that more than half of the patients died within 48 months. This information contributes to advances in the treatment of triple-negative breast tumors in Brazil, especially within the scope of the public health system, by highlighting and filling specific knowledge gaps. We also consider that these results may contribute to reflections on the panorama of the treatment of triple-negative breast tumors, as well as to gaining understanding of how the emergence of new technologies can impact the clinical outcomes presented by these patients and how the appropriate management of patients, within the scope of the public health system, can contribute to therapeutic effectiveness and efficiency, especially in contexts where access to innovative technologies is limited.

## Data Availability

Not available.
