# Supplementary material for: Use of medications in women with triple-negative breast cancer between 2018 and 2019 in a Brazilian public hospital: a retrospective study
Source: Epidemiol Serv Saude. 2025 Feb 21;34:e20240180. doi: 10.1590/S2237-96222025v34e20240180.en (PMC11845119; doi:10.1590/S2237-96222025v34e20240180.en)
Supplement: Translation

# Translation: Utilização de medicamentos em mulheres com câncer de mama triplo negativo tratadas entre 2018 e 2019 em um hospital público brasileiro: estudo descritivo

**Authors:** Monique Cristine da Silva Pires, Mario Jorge Sobreira-da-Silva, Patrícia Portella de Araújo, Maely Peçanha Favero Retto

**Affiliations:** 1 Instituto Nacional de Câncer, Programa de Residência Multiprofissional em Oncologia, Rio de Janeiro, RJ, Brasil; 2 Instituto Nacional de Câncer, Coordenação de Ensino, Rio de janeiro, RJ, Brasil; 3 Instituto Nacional de Câncer, Serviço de Farmácia, Rio de Janeiro, RJ, Brasil

**Keywords:** Neoplasias de Mama Triplo Negativas, Sistema Único de Saúde, Terapia Neoadjuvante, Quimioterapia Adjuvante, Farmacoepidemiologia.

## Abstract

**Objetivo:**

Descrever o perfil de utilização de medicamentos em mulheres com câncer de mama triplo negativo tratadas entre 2018 e 2019 em um hospital público brasileiro.

**Métodos:**

Estudo descritivo e retrospectivo, com dados obtidos em Registro Hospitalar de Câncer e de prontuários físicos e eletrônicos de um hospital público referência no tratamento de câncer, no Rio de Janeiro. Foram realizadas análises descritivas e análises de tempo até falha terapêutica e de sobrevida global, através do método de Kaplan Meier.

**Resultados:**

Das 176 pacientes, 39,0% tinham idade inferior a 50 anos e 47,7% foram diagnosticadas em estágio avançado. Identificou-se emprego de 12 esquemas de quimioterapia, com finalidade neoadjuvante ou adjuvante, para o tratamento do câncer de mama triplo negativo. O esquema terapêutico mais utilizado incluiu doxorrubicina, ciclofosfamida e taxano (docetaxel ou paclitaxel). Após 180 dias, 76,1% das pacientes permaneciam no tratamento inicial. O tempo médio até a falha terapêutica foi de 7,6 meses para as que seguiram o esquema principal. A mediana de sobrevida global foi de 34 meses, e 55,7% das pacientes faleceram até o final do período de seguimento (48 meses).

**Conclusão:**

Os resultados evidenciaram que o tratamento com doxorrubicina, ciclofosfamida e taxano (docetaxel ou paclitaxel) foi o mais utilizado nas pacientes analisadas, que o tempo médio de falha com este esquema terapêutico foi inferior a um ano e que mais da metade das pacientes foi a óbito em até quatro anos após o diagnóstico.

**Aspectos éticos**

A presente pesquisa respeitou os princípios éticos, obtendo os seguintes dados de aprovação:

Comitê de ética em pesquisa: Instituto Nacional de Câncer

Número do parecer: 5.970.438

Data de aprovação: 28/3/2023

Certificado de apresentação de apreciação ética: 67370523.6.0000.5274

Termo de consentimento: Dispensado

## Introdução

O câncer de mama é o tipo mais comum entre as mulheres em todo o mundo, correspondendo a apro- ximadamente 23,8% nessa população em 2022 ^1^. No Brasil, desconsiderando-se o câncer de pele não melanoma, é o tumor mais frequente, com previsão de 74 mil novos casos anuais até 2025 ^2^.

Dentre os cânceres mamários, o tumor de mama triplo negativo, definido pela ausência de receptores hormonais e não amplificação do gene do receptor do fator de crescimento epidermal humano 2, se distingue pela sua agressividade e prognóstico desfavorável ^3^. Essa caracterização o torna inapto para tratamentos hormonais e direcionados, limitando as opções te- rapêuticas, principalmente, o uso de quimioterapia sistêmica, sendo este um desafio clínico ^4^ ^,^ ^5^.

Na última década, houve um avanço significativo nas pesquisas voltadas ao desenvolvimento de novos tratamentos para o tumor de mama triplo negativo, com resultados promissores com o uso de imunote- rapia e terapias-alvo ^6^ ^,^ ^7^ ^,^ ^8^. Diretrizes internacionais destacam que, para alcançar melhores desfechos, pode ser necessário combinar terapias inovadoras à quimio- terapia sistêmica convencional em fases distintas do tratamento inicial ^9^.

Contudo, no Brasil, a quimioterapia sistêmica conti- nua sendo a principal opção disponível para tratamento dessa neoplasia no Sistema Único de Saúde - SUS ^10^ ^,^ ^11^. Estudos indicam que, além da demora na ado- ção de novas tecnologias, o acesso restrito a cuidados de saúde de qualidade, diagnóstico tardio e diferenças na disponibilidade de tratamentos inovadores contri- buem para desfechos variáveis entre as populações. Tais disparidades são ainda mais evidentes em países de renda baixa e média, como o Brasil, onde barreiras socioeconômicas agravam os desafios enfrentados pelas pacientes ^12^ ^,^ ^13^ ^,^ ^14^.

Devido à heterogeneidade das características do tumor de mama triplo negativo e aos medicamentos atualmente disponíveis no SUS, estabelecer um esque- ma terapêutico padrão é um desafio. Os dados sobre os procedimentos e protocolos adotados no tratamento dessa neoplasia no Brasil são escassos. Dessa forma, este estudo teve como objetivo caracterizar o perfil de utilização de medicamentos em mulheres com tumor de mama triplo negativo, com finalidade inicial neo- adjuvante e adjuvante, para compreender o padrão de tratamento adotado.

## Métodos

### Delineamento e contexto

Estudo descritivo e retrospectivo com pacientes diagnosticadas com tumor de mama triplo negativo, entre janeiro de 2018 e dezembro de 2019, tratadas com quimioterapia com finalidade inicial de tratamento neoadjuvante e adjuvante, em um hospital público referência em tratamento de câncer no Brasil.

### Participantes, amostra e critérios de elegibilidade

A população total no período foi constituída por 311 mulheres, maiores de 18 anos. Considerando-se um erro amostral de 5% e o intervalo de confiança de 95% (IC95%), estimou-se uma amostra mínima de 138 pacientes no estudo. Foram excluídas pacientes: gestantes e lactantes; com tumores concomitantes ou não primários de mama; que receberam tratamento sistêmico prévio em outra instituição; selecionadas para protocolos de pesquisa; cujo prontuário físico não foi localizado; que utilizaram hormonioterapia ou trastuzumabe e que apresentaram positividade hormonal ou do fator de crescimento epidermal 2 em algum momento do tratamento - apesar de inicialmente diagnosticadas com tumor de mama triplo negativo.

### Fonte de dados e variáveis

A base de dados utilizada para identificar a população do estudo foi o Integrador dos Registros Hospitalares de Câncer da instituição na qual o estudo foi realizado. Na sequência, realizou-se a seleção das participantes a partir das informações dos prontuários físico e eletrônico e do sistema de administração hospitalar da unidade.

As variáveis coletadas foram categorizadas em três eixos: dados sociodemográficos (idade inferior a 50 anos e igual ou superior a 50 anos, histórico fami- liar de câncer, raça/cor da pele, nível educacional e hábitos sociais); dados clínico-patológicos (escala de *performance*, subtipo histológico, grau histológico, expressão do marcador Ki-67, estadiamento clínico e patológico); dados terapêuticos (cirurgia, radioterapia, objetivo do tratamento e esquemas de quimioterapia realizados - incluindo doses, quantidade de ciclos realizados e medicamentos, além de motivo de troca de esquema terapêutico e linhas de tratamento paliativo, quando aplicável). A classificação de tumores malignos, conhecida como TNM (do inglês *tumour*, *lymph node*, *metastasis*) - *classification of malignant tumours* ^15^, foi utilizada para definir estadiamento clínico e patológi- co. Foram consideradas com estadiamento avançado aquelas com estadiamento clínico-patológico 3B, 3C e 4. Ademais, para a análise da sobrevida global, foi coletada a data de óbito.

### Organização e análise dos dados

Os dados foram organizados e analisados com uso do *software* Microsoft Office Excel® 365. Calculou-se a frequência das variáveis categóricas e as medidas de tendência central e dispersão das variáveis contínuas. Analisaram-se a distribuição das pacientes, ao longo de múltiplos esquemas de quimioterapia utilizados, e motivos de descontinuação do tratamento medicamen- toso. O tempo total de seguimento das pacientes foi de 48 meses, considerando-se a data de diagnóstico.

O tempo (em meses) para finalização dos esquemas utilizados como primeira linha foi estimado utilizando-se as datas inicial e final do tratamento. Estabelecido esse parâmetro, as transições das linhas de tratamento foram analisadas através do software Python versão 3.12.1, em 30, 60 e 180 dias.

Ao se identificar o esquema mais utilizado na primeira linha de terapia inicial, foi analisado o intervalo médio decorrido entre o início e a falha do tratamento, em 24 meses, usando-se o método de Kaplan-Meier ^16^. Foram considerados falhas: uso da linha subsequente com finalidade paliativa, óbito e cuidados paliativos exclusivos. A sobrevida global em 48 meses foi estimada a partir da data de diagnóstico. Nos casos em que ocorreu perda de seguimento, a censura aconteceu no momento do último registro clínico no prontuário da instituição. Para essas análises, foi utilizado o software JAMOVI® versão 2.3.28 ^17^ ^,^ ^18^.

## Resultados

Foram recrutadas 238 mulheres, sendo 62 excluídas por: ausência de registro de tratamento sistêmico (n=24; 38,7%); exibirem positividade hormonal e/ou utilizarem hormonioterapia (n=9; 14,5%); história de tratamento sistêmico em outra instituição (n= 8; 12,9%); história de quimioterapia prévia para outros tumores e/ou tumor de mama não primário (n=8; 12,9%); exibirem positividade para o fator de crescimento epidermal humano 2 e/ou utilização de trastuzumabe (n=5; 8,1%); apresentarem tumores concomitantes (n=4; 6,5%); participação em protocolos de pesquisa (n=3; 4,8%); e por não localização do prontuário físico (n=1; 1,6%).

Das 176 participantes da pesquisa, 85 (48,3%) foram diagnosticadas em 2018. A mediana de idade foi de 54 (min=24; máx=90) anos, sendo 39,0% com idade infe- rior a 50 anos. Do total, 77,8% se autodeclaravam não brancas e 50,6% possuíam apenas o ensino fundamental.

O subtipo tumoral mais frequente era o carcinoma ductal invasivo (95,5%), e, no momento do diagnóstico, a maioria apresentava performance status 1 (55,1%), grau histológico tipo II (50,0%), Ki 67 ≥14% (97,0%) e estadiamento avançado (47,7%). Das que fizeram cirurgia (n=136), 54,4% realizaram mastectomia radical. Um total de 67,6% das mulheres realizou radioterapia adjuvante. A Tabela 1 apresenta o perfil das pacientes analisadas.

**Tabela 1:** Características sociodemográficas e clínicas das pacientes com câncer de mama triplo negativo com finalidade de tratamento neoadjuvante ou adjuvante (n=176)

Variables	n (%)
**Histórico familiar de neoplasia**	
Sim	78 (44,3)
Não	98 (55,7)
**Raça/cor da pele**	
Não branca	137 (77,8)
Branca	39 (22,2)
**Escolaridade**	
Ensino fundamental	89 (50,6)
Ensino médio	65 (36,9)
Ensino superior	18 (10,2)
Ausência de informação	4 (2,3)
**Idade**	
18-49 anos	67 (39,0)
≥50 anos	109 (61,0)
Performance Status	
0	64 (36.4)
1	97 (55.1)
2	8 (4.5)
3	4 (2.3)
4	1 (0.6)
Ausência de informação	2 (1.1)
**Subtipo histológico**	
Carcinoma ductal invasivo	168 (95,5)
Carcinoma lobular invasivo ou outros	8 (4,6)
**Grau histológico**	
1	1 (0,6)
2	88 (50,0)
3	86 (48,8)
Ausência de informação	1(0,6)
**Expressão do marcador Ki 67**	
>14%	170 (97,0)
<14%	6 (3,0)
**Estadiamento**	
1A	7 (4,0)
2A	23 (13,1)
2B	31 (17,6)
3A	29 (16,5)
3B	75 (42,6)
3C	6 (3,4)
4	3 (1,7)
Ausência de informações	2 (1,1)
**Cirurgia**	
Sim	136 (77,3)
Não	40 (22,7)
**Tipo de cirurgia**	
Mastectomia radical	74 (42,0)
Segmentectomia	36 (20,5)
Mastectomia simples	25 (14,2)
Cirurgia realizada em outro hospital	1 (0,6)
Não realizou cirurgia	40 (22,7)
**Radioterapia**	
Sim	119 (67,6)
Não	57 (32,4)

Identificaram-se 12 esquemas de quimioterapia com finalidade neoadjuvante (10,8% dos casos) ou adjuvan- te (89,2% dos casos) para o tratamento do tumor de mama triplo negativo. A combinação entre doxorrubi- cina, ciclofosfamida e taxano (docetaxel ou paclitaxel) correspondeu ao principal esquema terapêutico utili- zado (80,7%). Os esquemas menos frequentes foram doxorrubicina (0,6%), docetaxel (0,6%) e a combinação entre fluoruracila, metotrexato e ciclofosfamida (0,6%).

No total, 23 pacientes seguiram para a segunda linha de quimioterapia com finalidade curativa, sendo tratadas com: carboplatina e paclitaxel (43,0%), paclitaxel (26,0%) capecitabina (9,0%), carboplatina (4,4%), ciclofosfamida e taxano (4,4%), gencitabina e cisplatina (4,4%), doceta- xel (4,4%) e fluoruracila, metotrexato e ciclofosfamida (4,4%). Na terceira linha neoadjuvante, cisplatina foi o medicamento de escolha para tratar duas pacientes. A distribuição das pacientes, ao longo de sucessivas linhas de quimioterapia, e os motivos para descontinuidade do tratamento são apresentados na Figura 1.

**Figura 1:** Distribuição de pacientes com câncer de mama triplo negativo ao longo de múltiplos esquemas de quimioterapia e motivos de descontinuação do tratamento medicamentoso

As motivações registradas nos prontuários para aplica- ção de múltiplos esquemas com finalidade neoadjuvante ou adjuvante foram: baixa efetividade terapêutica; pre- sença de lesões volumosas que dificultavam a realização de procedimentos cirúrgicos; condição clínica das pa- cientes; e progressão da doença.

Observou-se que, após uso do primeiro esquema terapêutico com finalidade curativa, 82 (46,6%) partici- pantes seguiram utilizando tratamento medicamentoso. Destas, 71,9% apresentaram progressão de doença e iniciaram uso de quimioterapia paliativa. Os motivos de descontinuação foram: resposta terapêutica -pacientes seguiram para acompanhamento de medicos mastologistas, em tratamentos que não envolviam a quimioterapia sistêmica; ausência de proposta tera- pêutica ou condições clínicas impeditivas - para essas, optou-se por empregar cuidados paliativos exclusivos ou cuidados de fim de vida.

A Figura 2 mostra que, no período de 30 dias após início do tratamento, apenas uma paciente havia migrado para uma segunda linha de tratamento neoadjuvante. Observa-se que, em até 60 dias, 22 pacientes iniciaram o uso de esquemas terapêuticos com finalidade paliativa, e registraram-se dois óbitos. Após 180 dias, 76,1% das pacientes permaneciam realizando o tratamento inicial.

**Figura 2:** Diagrama de Sankey com as alterações terapêuticas, em 30, 60 e 180 dias, das pacientes com câncer de mama triplo negative

Quando analisada a falha terapêutica em até 24 meses, verificou-se perda de seguimento de duas pa- cientes. Das 174 restantes, 70 (40,2%) apresentaram falhas relacionadas aos seguintes motivos: 55 (78,6%) utilizaram linha terapêutica subsequente com finalidade paliativa; 11 (15,7%) foram a óbito; e quatro (5,7%) foram encaminhadas para cuidados paliativos exclusivos. Entre as 70 pacientes, 55 (78,6%) tinham usado o esquema doxorrubicina, ciclofosfamida e taxano, com tempo médio de falha de 7,6 meses, em 24 meses (Figura 3).

**Figura 3:** Análise de temporalidade entre início do tratamento e falha do esquema terapêutico mais utilizado,composto por doxorrubicina, clofosfamida e taxano, no tratamento do câncer de mama triplo negativo com finalidade neoadjuvante e adjuvante, em 24 meses (n=55)

Durante o período de tratamento sistêmico, registrou--se o óbito de 56 pacientes (31,8%). Adicionalmente, 85 pacientes (48,3%) progrediram para a fase de tratamento não sistêmico. Ao término do período de seguimento, temos que: 60 (70,6%) permaneciam sob acompanha- mento da mastologia, sem necessidade de retorno para terapia sistêmica adicional; 15 (17,6%) foram a óbito; e cinco (5,9%) necessitaram ser reencaminhadas para terapia sistêmica. Observou-se, ainda, que duas (2,3%) participantes apresentaram progressão de doença sem indicação de tratamento medicamentoso, uma (1,2%) recebeu alta e foi encaminhada para acompanhamento na unidade de saúde de atenção primária, uma (1,2%) foi a óbito por covid-19 e uma (1,2%) foi internada em ambiente hospitalar com suporte não invasivo, sem registro posterior à internação.

Constatou-se que, após 48 meses, faleceram todas as pacientes que haviam sido direcionadas para cuidados de fim de vida, cuidados paliativos exclusivos, radiote- rapia paliativa, que optaram por não prosseguir com tratamento farmacológico, ou foram consideradas fora de proposta terapêutica. A Figura 4 mostra a sobrevida global em 48 meses. Observou-se sobrevida mediana de 34 meses, e que apenas 43,2% das pacientes analisadas permaneciam vivas ao final do seguimento. A perda de seguimento, ao final do estudo, foi de quatro pacientes, por ausência de registro clínico superior a 24 meses.

**Figura 4:** Análise de sobrevida global das mulheres com câncer de mama triplo negativo em 48 meses

## Discussão

Constatou-se que o esquema terapêutico mais utili- zado na primeira linha de tratamento foi a combinação doxorrubicina, ciclofosfamida e taxano, e que até três linhas de tratamento com finalidade curativa foram empregadas. Contudo, uma importante proporção de pacientes precisou fazer tratamento com finalidade paliativa após a primeira linha de tratamento curativo.

O tempo médio de falha do tratamento com doxor- rubicina, ciclofosfamida e taxano foi de 7,6 meses, a sobrevida global mediana foi de 34 meses a partir do diagnóstico, e, ao final do período de seguimento, menos da metade das pacientes permaneceram vivas.

A análise do perfil sociodemográfico e clínico demonstrou similaridade aos achados da coorte re- trospectiva conduzida de junho de 2008 a janeiro de 2009, que analisou mulheres diagnosticadas nos anos de 2001 (n=2.198) e 2006 (n=2.714) com subtipos distintos de câncer de mama no Brasil ^19^. No entanto, cabe destacar a significativa prevalência de mulheres jovens, com baixa escolaridade e raça/cor da pele não branca, no contexto em que o câncer se manifesta como questão de ampla relevância, englobando dimensões de saúde, sociais e econômicas ^1^, visto que o tumor de mama triplo negativo é conhecido por acometer mulheres jovens e apresentar pior prognóstico, com- parado a outros tipos de tumores da mama ^20^ ^,^ ^21^.

Estudos recentes apontam que o pior prognóstico pode não estar associado apenas às características biológicas do tumor. Diferentes autores investigaram a influência das disparidades sociais nos desfechos clínicos de mulheres com esse tipo de tumor e demons- traram que a raça/cor da pele não branca, privação socioeconômica e estresse social estavam associados à falta de acesso aos cuidados em saúde e pior sobre- vida global ^12^ ^,^ ^13^ ^,^ ^14^ ^,^ ^22^. Isso evidencia que os aspectos socioeconômicos podem estar relacionados a piores desfechos clínicos em pacientes com tumor de mama triplo negativo.

A distribuição das participantes pelas progressivas linhas de tratamento apontou para rápida progres- são da doença após a primeira linha de tratamento neoadjuvante e adjuvante, e necessidade de início de tratamento paliativo. Os dados revelam ainda que, após resposta insatisfatória ao regime composto por doxorrubicina, ciclofosfamida e taxano, a combinação de gencitabina e cisplatina emergiu como a opção mais recorrente, sendo este um esquema de escolha para tratamento do tumor de mama triplo negativo metas- tático e considerado seguro para pacientes tratadas com outras linhas de quimioterapia, quando em baixas doses ^23^ ^,^ ^24^.

A análise da taxa de falha em 24 meses para o regime terapêutico doxorrubicina, ciclofosfamida e taxano reve- lou considerável inefetividade após uso da primeira linha de tratamento. É importante destacar que os regimes terapêuticos empregados no tratamento das pacientes deste estudo foram baseados em práticas internas, alinhadas às sugestões de recomendações internacionais mais atualizadas para o tratamento do tumor de mama triplo negativo. Contudo, tais regimes eram dissonantes das diretrizes diagnósticas e terapêuticas vigentes em 2018 no sistema público de saúde brasileiro ^10^, que recomendavam um arsenal terapêutico ainda mais res- tritivo para o tratamento neoadjuvante ou adjuvante do tumor de mama triplo negativo. Nesse sentido, é possível pressupor que a adoção de esquemas terapêuticos desatualizados, previstos nas diretrizes, poderia resultar em desfechos ainda mais desfavoráveis no tratamento dessas pacientes.

A análise de sobrevida global em 48 meses de- monstrou que, apesar do incremento de atualizações advindas de diretrizes internacionais - que culminou no tratamento mais moderno frente às recomendações do Ministério da Saúde -, o que tem sido ofertado para tratar essas mulheres pode não estar sendo suficiente para a obtenção de melhores desfechos, diante de outras possibilidades terapêuticas. Ou, ainda, que o tratamento pode estar sendo iniciado por essas pa- cientes em tempo inoportuno. Estudos demonstram que o acesso ao tratamento do câncer de mama, no sistema público de saúde do Brasil, não ocorre con- forme o preconizado, e que as dificuldades podem ser exacerbadas quando se refere à população mais jovem ^25^ ^,^ ^26^.

O estudo retrospectivo que incluiu 8.601 mulheres e avaliou o papel da quimioterapia no câncer de mama triplo negativo no estágio IA, entre 2010 e 2019, de- monstrou que o uso de quimioterapia foi associado à melhora na sobrevida das pacientes. Considerando-se que, no presente estudo, a maior parte das pacien- tes acessou a atenção especializada com doença em estadiamento avançado, o manejo mais adequado dessas pacientes pela rede de saúde, e o consequente início precoce do tratamento, poderiam culminar em desfechos mais favoráveis.

Sugere-se que a implementação eficaz de estratégias de gestão no sistema público de saúde brasileiro possa resultar no direcionamento oportuno das pacientes para serviços de atenção especializada, assegurando que a aplicação de tecnologias já existentes na rede de cui- dados seja suficiente para proporcionar um tratamento mais efetivo. Além disso, a incorporação de novas tecnologias deve ser cogitada, mas não em detrimento da otimização de fluxos de encaminhamento e uso de recursos já existentes, de modo a se garantir que o emprego de medicamentos mais onerosos financeira- mente seja feito de maneira otimizada e sustentável, visto que o custeio dos mesmos é desafiador para os sistemas de saúde^28^.

No que concerne ao estudo de utilização de medi- camentos, algumas limitações devem ser consideradas, entre as quais, o uso de fontes de dados como prescri- ções médicas isoladas ou em prontuário e a coleta e consequente uniformização de dados sobre os medica- mentos ^29^. Para minimizar incertezas sobre os dados obtidos por estas fontes de dados, o presente estudo confrontou os dados de prescrição e/ou prontuário físico com os registrados nos sistemas informatizados do hospital.

Os resultados evidenciaram que o esquema te- rapêutico mais utilizado, em cenário neoadjuvante e adjuvante, foi a combinação doxorrubicina, ciclo- fosfamida e taxano, com tempo médio até a falha terapêutica de 7,6 meses, e que mais da metade das pacientes foi a óbito em 48 meses. Tais informações contribuem para o avanço no tratamento do tumor de mama triplo negativo no Brasil, sobretudo no âmbito do sistema público de saúde, ao serem evidenciadas e preenchidas lacunas específicas de conhecimento. Considera-se também que tais resultados poderão co- laborar para reflexões sobre o panorama do tratamento do tumor de mama triplo negativo, bem como para se compreender como o surgimento de novas tecnologias pode impactar os desfechos clínicos apresentados por essas pacientes e como a gestão adequada das pacientes, no âmbito do sistema público de saúde, pode contribuir para a efetividade e eficiência terapêutica, sobretudo em contextos onde o acesso a tecnologias inovadoras é limitado.

## Data Availability

Não disponível.
